# Machine Learning Models of Acute Kidney Injury Prediction in Acute Pancreatitis Patients

**DOI:** 10.1155/2020/3431290

**Published:** 2020-09-29

**Authors:** Cheng Qu, Lin Gao, Xian-qiang Yu, Mei Wei, Guo-quan Fang, Jianing He, Long-xiang Cao, Lu Ke, Zhi-hui Tong, Wei-qin Li

**Affiliations:** ^1^Surgical Intensive Care Unit (SICU), Department of General Surgery, Jinling Hospital, Medical School of Nanjing University, Nanjing, China; ^2^Surgical Intensive Care Unit (SICU), Department of General Surgery, Jinling Clinical Medical College of Southeast University, Nanjing, China; ^3^Electrical Engineering School of Southeast University, China; ^4^Institute for Hospital Management of Tsinghua University, Shenzhen, China

## Abstract

*Background*. Acute kidney injury (AKI) has long been recognized as a common and important complication of acute pancreatitis (AP). In the study, machine learning (ML) techniques were used to establish predictive models for AKI in AP patients during hospitalization. This is a retrospective review of prospectively collected data of AP patients admitted within one week after the onset of abdominal pain to our department from January 2014 to January 2019. Eighty patients developed AKI after admission (AKI group) and 254 patients did not (non-AKI group) in the hospital. With the provision of additional information such as demographic characteristics or laboratory data, support vector machine (SVM), random forest (RF), classification and regression tree (CART), and extreme gradient boosting (XGBoost) were used to build models of AKI prediction and compared to the predictive performance of the classic model using logistic regression (LR). XGBoost performed best in predicting AKI with an AUC of 91.93% among the machine learning models. The AUC of logistic regression analysis was 87.28%. Present findings suggest that compared to the classical logistic regression model, machine learning models using features that can be easily obtained at admission had a better performance in predicting AKI in the AP patients.

## 1. Introduction

Acute pancreatitis (AP) is an inflammatory abnormal condition of the exocrine pancreas, and most AP patients have mild disease courses and obtain recovery within one week [1]. There are about 20% of patients that will develop severe complications such as persistent organ failure and systemic inflammatory response syndrome (SIRS). Acute kidney injury (AKI) has long been recognized as a common and important complication of AP, and the incidence is as high as 10%-42% [2, 3]. Furthermore, AP patients concomitant with AKI suffer from a poor prognosis with a mortality of 25%-75% [4–7]. Hence, the early identification and timely management of AKI in AP patients seem very important. However, it is difficult to identify renal injury early depending on traditional indicators, and the main reason lies in that when there is an increase in serum creatinine (SCr) or a decrease in urine output, kidney damage has already occurred unstoppably [8].

Previous studies have identified a series of risk factors for predicting AKI, including triglyceride levels, age, male sex, procalcitonin, hypoxemia, abdominal compartment syndrome, and some biomarkers [9], and developed several AKI prediction models using classical regression methods [10–12]. However, their predictive performance was rarely reported regarding the area under the receiver operating characteristic curve (AUROC), the primary measure of the prediction model [13]. Furthermore, the classical logistic regression model is sensitive to the multicollinearity of independent variables, which makes the model easy to underfit and far from accurate. Recently, artificial intelligence applications have been gradually implemented in the medical field by using machine learning [14–16], having excellent performance in predicting complications compared to logistic regression analysis. Unsupervised learning and supervised learning are two types of machine learning used widely. Unsupervised learning such as random forest [17] and classification trees [18] allows the model to work on its own to discover information and mainly deals with the unlabeled data. Supervised learning such as extreme gradient boosting [19] learns from labeled training data and predicts outcomes for unforeseen data. However, there are few studies using machine learning approaches to predict acute kidney injury in AP patients.

Therefore, in this study, we aimed to develop AKI predictive models for AP patients by using different machine learning algorithms, mainly constituted of classification and regression tree (CART), random forest (RF), support vector machine (SVM), and extreme gradient boosting (XGBoost), as well as comparing the their predictive performances with those of the classical multivariable logistic regression (LR) methods.

## 2. Methods

### 2.1. Patients

We performed a retrospective observational study of AP patients admitted to the Center of Severe Acute Pancreatitis (CSAP) of Jinling Hospital, Nanjing, China, from January 2014 to January 2019. The center is a tertiary center for acute pancreatitis located in eastern China. Patients who met the following criteria were included: (1) diagnosis of AP and (2) admission to our department within one week after the disease onset. Patients who were older than 75 or younger than 18 already developed AKI before admission and suspected of chronic pancreatitis, pancreatic tumors, pancreatic trauma, and pregnancy were excluded to minimize bias. All the data were retrieved from a prospectively collected electronic database with the approval of the Acute Pancreatitis Database Management Committee. Informed consent from individuals was waived due to the retrospective, observational, and anonymous nature of the current study.

### 2.2. Definition

AP (ICD-10, K85) was diagnosed according to the definition in the 2012 revision of the Atlanta classification [20]. Acute kidney injury (AKI) (ICD-10: N17) was diagnosed and staged using the Kidney Disease: Improving Global Outcomes (KDIGO) criteria based on serum/plasma creatinine and urine output [8]. And the patient meeting the diagnosis during the whole hospitalization of AP is calculated into the AKI group. Alcohol abuse (ICD-10, F10) and smoking (Z72.0, Z86.43, and Z87.891) were identified using relevant diagnostic codes.

### 2.3. Data Collection

We collected data on demographic characteristics, previous medical history, physical examination, laboratory examination, and therapeutic treatments of each patient. Based on previous studies, we selected 23 possible risk factors for predicting AKI, including etiology, demographic data (age, gender, smoking, and alcohol abuse), body mass index (BMI), hypertension, intra-abdominal pressure (IAP), disease severity scores (APACHE II), acute respiratory distress syndrome (ARDS), and laboratory examination (amylase, lipase, triglyceride (TG), cholesterol, white blood cells (WBC∗10^9^), c-reactive protein (CRP), interleukin-6 (IL-6), procalcitonin (PCT), total bilirubin (TBIL), alanine aminotransferase (ALT), hemoglobin (Hb), platelet (PLT), and prothrombin time (PT)). All of the data were available from the hospitalized patient electronic medical record system within 24 h after admission. However, the values of serum IL-6 levels were not complete (240 out of 334 total patients), so we filled the lost value with the mean of the remaining data.

### 2.4. Statistical Analysis

The population characteristics are presented using medians and interquartile ranges (IQR) for continuous variables and count and percentages for the dichotomous variables. For continuous variables, we used the Kolmogorov–Smirnov test to analyze the normalization of the distributed data and used Mann–Whitney *U* tests to analyze nonnormally distributed data. A *p* value < 0.05 was taken as statistically significant.

Prior to developing predictive models, the data collected were divided into 70% of the training dataset and 30% of the test dataset. The training dataset was used for developing predictive models using machine learning and logistic regression algorithms. The parameters of the models were continuously adjusted using tenfold cross-validation to reduce the chances of overfitting, and then, the final performance of each model was validated and compared in the test dataset. The area under the receiver operating characteristic curve (AUC), sensitivity, specificity, and accuracy were adopted as the comparative measure between different models.

The modeling and statistical analyses were performed using Sklearn package version 0.19 (https://scikit-learn.org/stable/) and Python programming software version 3.6 (Python Software Foundation, http://www.python.org/).

### 2.5. Logistic Regression Algorithms and Machine Learning Algorithms

#### 2.5.1. Logistic Regression (LR)

**Figure 1:**
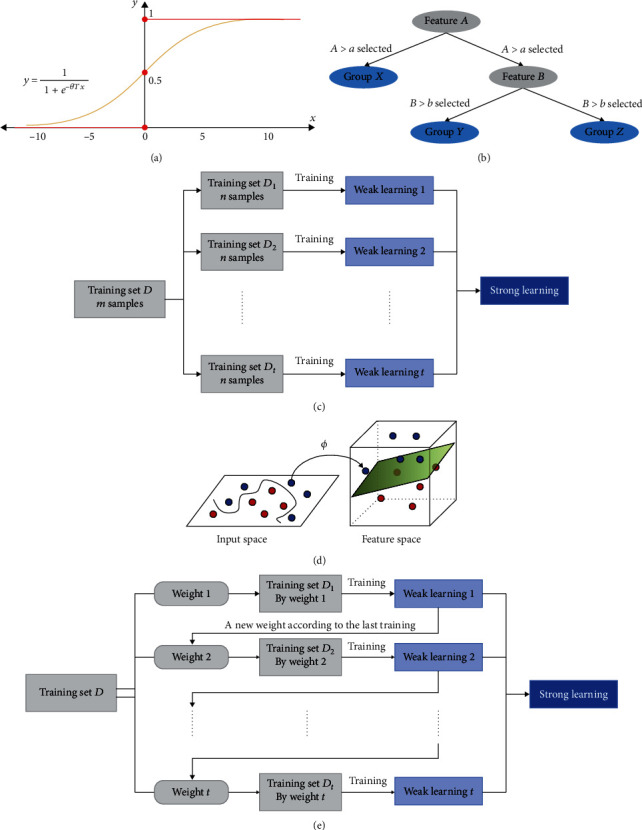
The graphic expression of different machine learning algorithms: (a) logistic regression, (b) classification and regression tree, (c) random forest, (d) support vector machine, and (e) extreme gradient boosting.

The logistic regression model is a discrete selection and generalized linear regression analysis model [21]. It has been widely used in medicine, industry, and other areas. It uses the sigmoid function to map the predicted value to a probability value on (0, 1) to help judge the result (Figure 1(a)). This model can be applied to both continuous and categorical independent variables.

#### 2.5.2. Classification and Regression Tree (CART)

The classification and regression tree [18] is a tree-like prediction model (Figure 1(b)). Each nonleaf node in the tree represents a feature value input by the model. The branch path under the node represents the possible attributes of the feature value. Each leaf node represents one or more samples, and the path taken from the root node to the leaf node represents the classification process of the sample. The decision tree itself has no specific requirements for the input eigenvalues and can be used for both numerical data (including continuous and discrete outcome) and logical or categorical data. The CART algorithm uses the Gini index to select the optimal feature. The Gini index represents the purity of the model, and its value is between 0 and 1.

#### 2.5.3. Random Forest (RF)

The random forest is an integrated classifier with multiple decision trees [17], which belongs to the bagging algorithm (Figure 1(c)). There is no dependency between the weak learners that can be generated in parallel and fitted. The outputs of the weak learners are combined (by mean, mode, etc.) as a model output. The random forest is an evolved version of the bagging algorithm which uses a CART decision tree as a weak learner.

#### 2.5.4. Support Vector Machine (SVM)

The support vector machine [22] is a supervised learning model applied to classification and regression problems. For linearly separable problems, the model constructs hyperplanes (sets) in a high-dimensional or infinite-dimensional space to separate samples; for linearly inseparable problems, the model chooses a suitable kernel function (*φ*) to map the samples to a high-dimensional space that is much higher than the original space dimension, so that the samples are linearly separable in the high-dimensional space (Figure 1(d)).

#### 2.5.5. Extreme Gradient Boosting (XGBoost)

The extreme gradient boosting (XGBoost) is an efficient system implementation of the Gradient Boosting Decision Tree (GBDT) algorithm, which belongs to the boosting algorithm [19, 23]. (1) Weak learner 1 is trained with initial weights from the training set, (2) the weights of the training samples are updated according to the learning error rate, (3) the weights of weak learner 1 are increased, (4) weak learner 2 will be trained with new weights, and this will be iterated until the number of weak learners reaches the specified number *T*, and (5) finally, a total of weak learners are combined to obtain the final strong learner (Figure 1(e)).

## 3. Results

In this study, we extracted 23 features, including 17 continuous variables (Table 1) and 6 dichotomous variables (Table 2) from 334 AP patients who were admitted within one week after the AP onset. Among the study patients, finally, 80 patients (23.95%) developed AKI during the whole hospitalization among whom 13 patients suffered from AKI stage 1, 37 patients from stage 2, and 30 patients from stage 3 according to the KIDGO criteria.

The results showed that in comparison with patients in the non-AKI group, patients who suffered from AKI had a higher incidence of ARDS (*p* < 0.001) and death (*p* < 0.001); higher BMI (*p* = 0.005), IAP (*p* < 0.001), and APACHE II scores (*p* < 0.001); and higher percentages of male sex (*p* = 0.005) and alcohol consumption (*p* = 0.020), together with significantly higher serum levels of CRP (*p* = 0.012), PCT (*p* < 0.001), and TBIL (*p* < 0.001). The serum levels of Hb (*p* = 0.006) and PLT (*p* < 0.001) in the AKI group are lower compared with those in the non-AKI group.

### 3.1. Predictive Effects of Different Models

**Figure 2:**
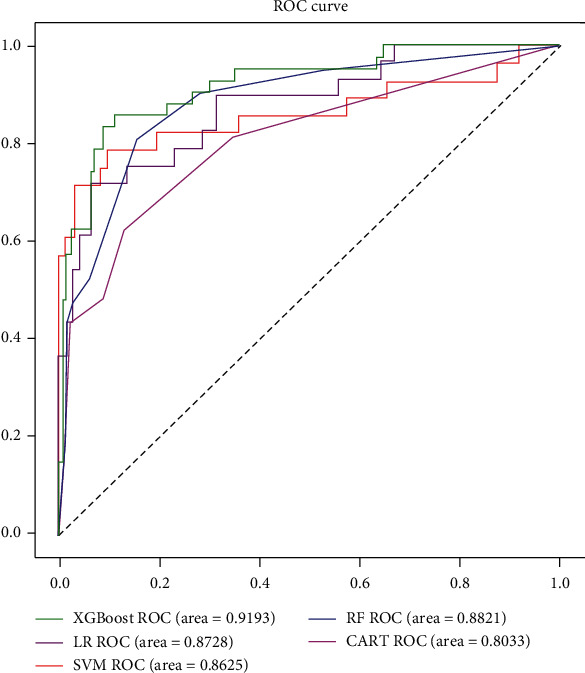
The receiver operating characteristic (ROC) curves of 5 different models in predicting AKI in AP patients after admission in the test dataset. Abbreviations: XGBoost: extreme gradient boosting; RF: random forest; SVM: support vector machine; LR: logistic regression; CART: classification and regression tree.

We generated five models, including LR (logistic regression), SVM (support vector machine), XGBoost (extreme gradient boosting), RF (random forest), and CART (classification and regression tree), to predict the development of AKI in AP patients after admission.  Figure 2 shows the performance of 5 different models in predicting AKI on the test dataset in terms of receiver operating characteristic (ROC) curves. The areas under ROC curves (AUC) demonstrated that the XGBoost model achieved the best predictive effects for AKI with an AUC of 0.9193 compared with other models. Taking the LR model as a reference, the XGBoost model and RF model outperformed it in predicting AKI while the SVM model and CART model failed as shown by AUC values.

Table 3 presents a set of detailed performance metrics for the 5 models. As to all of the five metrics, the XGBoost achieved the best performance with the highest AUC (0.9193), the highest sensitivity (0.6190), the highest specificity (0.8815), and the second-highest accuracy (0.8631). The ranks of feature importance in each model are listed in Table 4. As shown, APACHE II, IAP, and PCT rank the top three features contributing to the development of the prediction models for AKI in AP patients.

## 4. Discussion

Acute kidney injury (AKI) is a common complication of AP, and its incidence is 14%-43% [2, 3, 24]. According to relevant research reports, AKI developed by AP may be caused by the release of a large number of inflammatory mediators and cytokines, which lead to microcirculation disorders and tissue damage [25]. At the same time, hypercoagulability and SIRS may cause damage to renal tubules [26]. In this study, PCT is the second most important risk factor in the XGBoost. The clinical outcomes of AP patients complicated with AKI are extremely poor, and the mortality reported in the previous studies is up to 40-70% [6, 24]. Hence, it should be at the top of the priority list to identify high-risk patients and prevent their renal function from further deterioration.

We compared the performance of four machine learning models and the traditional logistic regression model to predict AKI in the early stage. The result showed that XGBoost achieved the best performance in predicting AKI in terms of the combined predictive performance and predictive stability. XGBoost is a scalable tree boosting system that is widely used by data scientists and provides state-of-the-art results on many problems. XGBoost helps to reduce overfitting compared to gradient tree boosting by only a random subset of descriptors in building a tree and is known as the “regularized boosting” technique. The balance between sensitivity and specificity for each of the algorithms should also be evaluated. In particular, XGBoost had higher specificity than sensitivity, meaning it is more prone to be correct in ruling out AKI than detecting it. Our results demonstrated that the XGBoost appears to be a very effective machine learning method in terms of specificity and accuracy.

We listed the features of the highest importance in the three best-performing models. The APACHE II score, IAP, PCT, and lipase turned out to be the top three most important features. The APACHE II score is a nonspecific scoring system, which is related to the severity and complications of AP [27, 28]. Previous studies found that the APACHE II score is an independent risk factor for AP complicated with AKI [29, 30]. The median APACHE II score of patients in the AKI group is much higher than that in the non-AKI group (18.28 vs. 9.87, *p* < 0.005). IAP is the most important feature in the XGBoost model, and previous studies showed that IAP is the independent risk factor for AKI [31–34]. Locally in the abdomen, intra-abdominal hypertension compresses and compromises blood flow in the renal parenchyma, vena cava, and renal vein. Increased IAP has a multitude of effects on the kidney through a series of mechanisms that result in a decrease in the glomerular filtration rate (GFR) with oliguria, which usually is the first clinical evidence of kidney impairment [35, 36]. Screening and intervention to decrease IAP and improve vital perfusion of the kidney are essential to minimize the negative effects [37].

Novel machine learning techniques are relatively free of these limitations of conventional statistical analysis and have demonstrated improved predictive performance compared to classical statistical methods, and machine learning has been used to predict AKI in some disease populations (e.g., severely burned patients and patients receiving liver transplants) and shows favorable performances [38, 39]. Compared with traditionally static predictive models, deep-learning techniques have the advantages in the ability to automatically learn the features and relationship of the readily available data [40], which makes the early prediction of AKI possible before the significant changes in classical indicators, for instance, creatinine and/or urine output. Earlier identification of renal injury with the easily obtained medical data at admission provides a “therapeutic window” for clinicians to take preventive measures to avoid further renal function damage.

Previous studies showed that early detection and treatment of AKI can help most patients recover renal function and reach a better clinical outcome [41, 42]. Therefore, it is particularly important to identify the risk factors and prognostic factors for acute pancreatitis with acute renal injury in the early stage, so as to develop a predictive model to help clinicians take preventive intervention measures and avoid renal function damage [43]. Our study provides a predictive model with machine learning algorithms that can give a better performance in predicting AKI of AP patients than the classical LR algorithm. A model using machine learning produced by our study may have a positive effect on the outcome of the AP patients.

Our study has several limitations. Firstly, our analysis used only a small number of cases from data derived from a single AP treatment center. There may be some differences in the performance of machine learning techniques when they are applied to a sample of a different institution with a different distribution of covariates. Secondly, the study does not use the models produced by the last 5 years in our center, in other centers, or in some open databases.

Compared to the classical logistic regression model, machine learning models (XGBoost and RF) using features that can be easily obtained at admission had a better performance in predicting AKI in the AP patients. Predictive models using machine learning algorithms may help clinicians predict AKI early and may prevent the renal function from further injury.

## Figures and Tables

**Table 1:** The continuous variable characteristics of AP patients.

No.	Variable code	Non-AKI group (*n* = 254)	AKI group (*n* = 80)	*p* values
	AKI stage (count, %)			
	Stage 1		13 (16.25%)	
	Stage 2		37 (46.25%)	
	Stage 3		30 (37.50)	
1	Age (year)	45.85 (37.00, 54.00)	46.86 (39.62, 51.55)	0.559
2	AMY (unit)	422.53 (77.00, 457.00)	773.33 (118.25, 840.75)	0.098
3	LPS (unit)	1132.58 (240.00, 1234.00)	1750.73 (330.25, 1442.25)	0.087
4	TG (mmol/L)	5.76 (1.00, 5.70)	7.034625 (2.30, 6.89)	0.300
5	Chol (mmol/L)	4.841 (3.08, 5.60)	4.58 (2.30, 6.89)	0.533
6	WBC (∗10^9^/L)	12.54 (9.10, 14.80)	12.02 (8.35, 14.25)	0.412
7	CRP (mg/L)	155.54 (94.10, 213.10)	189.22 (147.35, 236.90)	0.012
8	IL-6 (pg/mL)	156.96 (44.35, 161.15)	283.66 (105.50, 174.50)	0.08
9	PCT (*μ*g/L)	2.17 (0.21, 1.99)	13.61 (1.71, 16.98)	<0.001
10	TBIL (*μ*mol/L)	24.51 (14.60, 28.70)	45.21 (18.85, 51.53)	<0.001
11	ALT (U/L)	65.75 (20.00, 59.00)	62.35 (22.25, 65.50)	0.82
12	Hb (g/L)	125.39 (109.00, 141.00)	114.75 (90.00, 137.25)	0.006
13	PLT (∗10^9^/L)	174.89 (126.00, 215.00)	128.98 (84.50, 179.75)	<0.001
14	PT (s)	13.56 (12.20, 13.70)	13.59 (12.23, 14.48)	0.971
15	BMI	25.37 (23.00, 27.00)	26.60 (24.60, 29.08)	0.005
16	APACHE II	9.87 (7.00, 13.00)	18.28 (12.00, 17.00)	<0.001
17	IAP (mmHg)	7.26 (5.00, 10.00)	14.11 (12.00, 17.00)	<0.001

Abbreviations: AMY: serum amylase; LPS: serum lipase; TG: triglycerides; Chol: cholesterol; WBC: white cell count; CRP: c-reactive protein; IL-6: interleukin-6; PCT: procalcitonin; TBIL: total bilirubin; ALT: alanine aminotransferase; Hb: hemoglobin; PLT: platelet; PT: prothrombin time; BMI: body mass index; APACHE II: Acute Physiology and Chronic Health Evaluation II; IAP: intra-abdominal pressure.

**Table 2:** The dichotomous variable characteristics and outcomes of AP patients.

No.	Variable code	Variable description	Non-AKI		AKI		*P* values
			Count	Percent (%)	Count	Percent (%)	
1	Gender	Male	157	61.57	62	77.50	0.005
2		Female	98	38.43	18	22.50	
3	Etiology	Biliary	126	49.41	31	38.75	0.094
4		Hyperlipidemic	105	41.18	44	55.00	0.030
5		Alcoholic	4	1.57	2	2.50	0.585
6		Other	20	7.84	3	3.75	0.134
7	Smoking		91	35.69	36	45.00	0.145
8	Alcohol abuse		60	23.53	28	35.00	0.020
9	Diabetes		58	22.75	17	21.25	0.780
10	Hypertension		66	25.88	30	37.50	0.059

	ARDS		33	12.9	55	61.10	<0.001
	Death		1	0.40	16	17.8	<0.001

Abbreviations: ARDS: acute respiratory distress syndrome; Death: death during the hospitalization; AKI: acute kidney injury.

**Table 3:** The detailed performance metrics for the 5 models.

	LR	CART	XGBoost	SVM	RF
AUC	0.8728	0.8033	0.9193	0.8625	0.8821
Sensitivity	0.6071	0.6190	0.6190	0.5357	0.4761
Specificity	0.8642	0.8333	0.8815	0.8488	0.8472
Accuracy	0.8614	0.7910	0.8631	0.8713	0.8452

Abbreviations: AUC: area under the receiver operating characteristic curve; LR: logistic regression; XGBoost: extreme gradient boosting; SVM: support vector machine; RF: random forest.

**Table 4:** The ranks of feature importance in XGBoost, RF, and LR for predicting AKI.

Rank	RF	XGBoost	LR
1	APACHE II	IAP	APACHE II
2	IAP	PCT	IAP
3	PCT	APACHE II	LPS
4	CRP	TBIL	TBIL
5	TBIL	TG	PCT

Abbreviations: RF: random forest; XGBoost: extreme gradient boosting; LR: logistic regression; APACHE II: Acute Physiology and Chronic Health Evaluation II; IAP: intra-abdominal pressure; PCT: procalcitonin; CRP: c-reactive protein; TBIL: total bilirubin; TG: triglycerides; LPS: serum lipase.

## Data Availability

The data in this study are available for other researchers to verify the results of our article, replicate the analysis, and conduct secondary analyses. Other researchers can send e-mails (e-mail address: njzyantol@hotmail.com) to contact us for obtaining our data.

## References

[1] Forsmark C. E., Vege S. S., Wilcox C. M. (2017). Acute pancreatitis. *The New England Journal of Medicine*.

[2] Ljutić D., Piplović-Vuković T., Raos V., Andrews P. (1996). Acute renal failure as a complication of acute pancreatitis. *Renal Failure*.

[3] Compañy L., Sáez J., Martínez J. (2003). Factors predicting mortality in severe acute pancreatitis. *Pancreatology*.

[4] Nasir K., Ahamd A. (2012). Clinical course of acute pancreatitis in chronic kidney disease patients in a single kidney center (PGTi) in Karachi. *The Arab Journal of Nephrology and Transplantation*.

[5] Petejova N., Martinek A. (2013). Acute kidney injury following acute pancreatitis: a review. *Biomedical Papers of the Medical Faculty of the University Palacky, Olomouc, Czech Republic*.

[6] Kes P., VuČIČEviĆ Ž., RatkoviĆ-GusiĆ I., Fotivec A. (2009). Acute renal failure complicating severe acute pancreatitis. *Renal Failure*.

[7] Lin H. Y., Lai J. I., Lai Y. C., Lin P. C., Chang S. C., Tang G. J. (2011). Acute renal failure in severe pancreatitis: a population-based study. *Upsala Journal of Medical Sciences*.

[8] Disease K. (2012). Kidney Disease: Improving Global Outcomes (KDIGO) acute kidney injury work group. *Kidney International*.

[9] Li H., Qian Z., Liu Z., Liu X., Han X., Kang H. (2010). Risk factors and outcome of acute renal failure in patients with severe acute pancreatitis. *Journal of Critical Care*.

[10] Wu J., Xu Z., Zhang H. (2019). Clinical study on the early predictive value of renal resistive index in acute kidney injury associated with severe acute pancreatitis. *Zhonghua Wei Zhong Bing Ji Jiu Yi Xue*.

[11] Chai X., Huang H. B., Feng G. (2018). Baseline serum cystatin C is a potential predictor for acute kidney injury in patients with acute pancreatitis. *Disease Markers*.

[12] Wu C., Ke L., Tong Z. (2014). Hypertriglyceridemia is a risk factor for acute kidney injury in the early phase of acute pancreatitis. *Pancreas*.

[13] Zou K. H., O’Malley A. J., Mauri L. (2007). Receiver-operating characteristic analysis for evaluating diagnostic tests and predictive models. *Circulation*.

[14] Gupta A., Liu T., Shepherd S., Paiva W. (2018). Using statistical and machine learning methods to evaluate the prognostic accuracy of SIRS and qSOFA. *Healthcare Informatics Research*.

[15] Le S., Hoffman J., Barton C. (2019). Pediatric severe sepsis prediction using machine learning. *Frontiers in Pediatrics*.

[16] Arefan D., Mohamed A. A., Berg W. A., Zuley M. L., Sumkin J. H., Wu S. (2019). Deep learning modeling using normal mammograms for predicting breast cancer risk. *Medical Physics*.

[17] Breiman L. (2001). Random Forests. *Machine Learning*.

[18] Quinlan J. R. (1986). Induction of decision trees. *Machine Learning*.

[19] Chen T., Guestrin C. XGBoost: a scalable tree boosting system.

[20] Banks P. A., Bollen T. L., Dervenis C. (2012). Classification of acute pancreatitis--2012: revision of the Atlanta classification and definitions by international consensus. *Gut*.

[21] Bewick V., Cheek L., Ball J. (2005). Statistics review 14: logistic regression. *Critical Care*.

[22] Cortes C., Vapnik V. (1995). Support-vector networks. *Machine Learning*.

[23] Friedman J. H. (2001). Greedy function approximation: A gradient boosting machine. *The Annals of Statistics*.

[24] Pupelis G. (2000). Renal failure in acute pancreatitis. Timing of dialysis and surgery. *Przeglad Lekarski*.

[25] Bonegio R., Lieberthal W. (2003). Role of apoptosis in the pathogenesis of acute renal failure. *Current Opinion in Nephrology and Hypertension*.

[26] Papachristou G. I. (2008). Prediction of severe acute pancreatitis: current knowledge and novel insights. *World Journal of Gastroenterology*.

[27] Banks P. A., Freeman M. L. (2006). Practice guidelines in acute pancreatitis. *The American Journal of Gastroenterology*.

[28] Larvin M. (1997). Assessment of severity and prognosis in acute pancreatitis. *European Journal of Gastroenterology & Hepatology*.

[29] Kim B. G., Noh M. H., Ryu C. H. (2003). A comparison of the BISAP score and serum procalcitonin for predicting the severity of acute pancreatitis. *The Korean Journal of Internal Medicine*.

[30] Jin Z., Xu L., Wang X., Yang D. (2017). Risk factors for worsening of acute pancreatitis in patients admitted with mild acute pancreatitis. *Medical Science Monitor*.

[31] Matthew D., Oxman D., Djekidel K., Ahmed Z., Sherman M. (2013). Abdominal compartment syndrome and acute kidney injury due to excessive auto-positive end-expiratory pressure. *American Journal of Kidney Diseases*.

[32] Al-Bahrani A. Z., Abid G. H., Holt A. (2008). Clinical relevance of intra-abdominal hypertension in patients with severe acute pancreatitis. *Pancreas*.

[33] De Waele J. J., Hoste E., Blot S. I., Decruyenaere J., Colardyn F. (2005). Intra-abdominal hypertension in patients with severe acute pancreatitis. *Critical Care*.

[34] Rosas J. M. H., Soto S. N., Aracil J. S. (2007). Intra-abdominal pressure as a marker of severity in acute pancreatitis. *Surgery*.

[35] Li W.-D., Jia L., Ou Y., Huang Y. X., Jiang S. M. (2013). Surveillance of intra-abdominal pressure and intestinal barrier function in a rat model of acute necrotizing pancreatitis and its potential early therapeutic window. *PLoS One*.

[36] Pupelis G., Austrums E., Snippe K., Berzins M. (2016). Clinical significance of increased intraabdominal pressure in severe acute pancreatitis. *Acta Chirurgica Belgica*.

[37] Goenka M. K., Goenka U., Afzalpurkar S., Tiwari S. C., Agarwal R., Tiwary I. K. (2020). Role of static and dynamic intra-abdominal pressure monitoring in acute pancreatitis. *Pancreas*.

[38] Tran N. K., Sen S., Palmieri T. L. (2019). Artificial intelligence and machine learning for predicting acute kidney injury in severely burned patients: a proof of concept. *Burns*.

[39] Lee H. C., Yoon S., Yang S. M. (2018). Prediction of acute kidney injury after liver transplantation: machine learning approaches vs. logistic regression model. *Journal of Clinical Medicine*.

[40] LeCun Y., Bengio Y., Hinton G. (2015). Deep learning. *Nature*.

[41] Tran D. D., Oe P. L., De Fijter C. W. H., Van der Meulen J., Cuesta M. A. (1993). Acute renal failure in patients with acute pancreatitis: prevalence, risk factors, and outcome. *Nephrology, Dialysis, Transplantation*.

[42] Shah S., Leonard A. C., Harrison K., Meganathan K., Christianson A. L., Thakar C. V. (2020). Mortality and recovery associated with kidney failure due to acute kidney injury. *Clinical Journal of the American Society of Nephrology*.

[43] Gajic O., Dabbagh O., Park P. K. (2011). Early identification of patients at risk of acute lung injury: evaluation of lung injury prediction score in a multicenter cohort study. *American Journal of Respiratory and Critical Care Medicine*.

